# Indents

**Published:** 1916-04

**Authors:** 


### INDENTS

**☞ Brief Articles of Technical or Scientific Value will receive notice and comment. Contributions invited.**

*The Interrelation Between Salivary and Gastric Digestion.*

Physiologists have long known that the starch-digesting enzyme present in the saliva of man is destroyed as soon as it is exposed to even small concentrations of "free" hydrochloric acid, or, in terms of modern chemical interpretation, hydrogen ions present in the stomach. From the standpoint of a useful performance on the part of the starch-digesting saliva, the ready inhibition of its effectiveness as a digestive agent was somewhat mystifying when it was first discovered; for the duration of amylolytic activity appeared to be restricted essentially to the very brief period during which the foods are retained in the mouth, masticated and swallowed. Subsequently it was ascertained that the actual sequence of events within the stomach does not compel an immediate mixing of the entering contents with the gastric juice which is being secreted. The mass that is swallowed in successive portions accumulates at first in the fundus of the stomach; and since in the absence of vigorous muscular movements in that region the contents are penetrated with great difficulty by the gastric secretion which is continually being poured out by the walls of the stomach, salivary digestion can actually proceed for a considerable time without serious interruption. In view of the rapidity with which the salivary enzyme can convert starch into soluble sugar, the preliminary digestion of carbohydrates can therefore usually be completed before destruction of the effective agent in the saliva takes place. Dr. Maxwell, of the physiologic laboratory at the University of Melbourne, has suggested a further function of the salivary enzyme which he believes to be of importance for the later digestive processes. It has long been known that many substances in suspension or in colloidal solution have the power of absorbing enzymes, thereby inhibiting their activity. Maxwell has found experimentally that although unboiled starch administered in the form of intact grains does not hinder the action of pepsin, peptic digestion may be delayed in the presence of colloidal starch solutions through absorption of the preteolytic enzyme. The time interval for the peptic digestion may, for example, be increased fourfold in the presence of a 2 per cent. starch solution. There is a stage in the progressive digestive disruption of the starch molecule at which the capacity of absorption of pepsin is lost. This is coincident with the appearance of dextrins, even before sugars are formed. In accord with the foregoing it is actually found that cooked farinaceous foods—rice, potato, bread, porridge, etc. —all hinder peptic digestion if they are not first subjected to the salivary digestion. The inhibition of peptic activity by carbohydrates like gum acacia is not prevented by a previous contact with saliva for the reason that they are not digested by it. The positive feature to which Maxwell has drawn attention has been summarized by the statement that the saliva of man, by virtue of its enzyme ptyalin or amylase, plays a considerable part in aiding gastric digestion by hydrolyzing colloidal starch which would otherwise absorb pepsin.—*Journal of American Medical Association.*

*Interpretation of Radiographs.* Dental radiography, in order to be of diagnostic value, must be carried out by radiographers to whom dental anatomy and pathology are an open book. The technique of radiography, and here we mean specifically the mechanics of radiography, is a relatively simple matter, at least one which does not demand of the individual a superlative degree of skill or anything akin to it. The interpretation of dental radiographs is altogether a different matter. Here the untrained mind in the process of disease etiology and disease evolution must find itself at bay to correctly interpret the shadow densities and a diagnosis on so flimsy a foundation must of necessity lead, in a series of cases, to serious complications. Cases have recently come to the writer's attention in which a definite diagnosis of pathologic conditions supposedly present in the mouth had been made by radiographers whose training in dental pathology and clinical dentistry was nil, in fact by men who perhaps know the mechanics of radiography as far as the production of pictures is concerned—pictures in which correct anatomic proportions are not always to be discerned—but with no medical or dental training whatsoever. This is truly appalling, especially because it is a reflection of the erroneous conception of radiographic diagnosis and of its possibilities when in the hands of the expert radiographer.

When some of these requisites are happily combined such valuable studies and conclusions as those of Dr. McCoy's are possible and irrefutable, otherwise the radiograph becomes a dangerous weapon, one which should be as strongly condemned as the product of the radiographer-pathologist should be praised.—Dr. Endelman in *Pacific Gazette.*

*Advertising.* In the various articles on dental advertising published in the *Digest* the past year, one point seems to have been missed. Suppose it were perfectly legitimate for all dentists to advertise and all dentists did so, what advantage would one have over another? If one were not capable of good advertising could he not revert to a professional advertisement writer and thus do as good advertising as the other fellow, and all advertising being equal would there not be a large expense thus added to the dental profession without any advantage to any one party? Any dentist of good morals, of fair workmanship, attentive to business, associating with only the best of associates and being conspicuous in public affairs need not even see wolf tracks within a hundred miles of his office or home, and a country crossroads may be his place of business. R. A. W. in *Dental Digest.*

*Rugae on Plates.* After flask is opened, wax removed, take suitable carving instruments and different sizes of ball burnishers, and carve rugae on plaster in the half containing teeth. Burnish tin-foil over this, pack and vulcanize. It takes only a few minutes, and you are well repaid for your trouble.—H. L. Entriken in *Digest.*

*A Short, Accurate Method of Adapting a Porcelain Crown.* Prepare root as desired, grind crown to approximately fit root, adjust post, mix synthetic porcelain as for filling, cover base of crown with the prepared porcelain, insert post in crown, dry root of tooth, place piece of tin foil over end of root to exclude secretions, place crown in position, holding in place until porcelain is partially set, remove crown and post intact, lay aside for about fifteen minutes until porcelain is thoroughly crystalized, then polish and set with cement.—A. M. Wilkes in *Dental Review.*

*Root Canal Filling—Two Methods.* "*First Method,* Dr. Callahans method: Dissolve 12 gr. resin (violin bow) in two drams of chloroform. This preparation is carried to the orifice of the canal with the dressing pliers, and pumped to the apex, followed by gutta-percha points to fill the canal.

"*Second Method,* by Dr. Buckley: It has been found that air can not be displaced by a solid or a semi-solid. It is therefore necessary to displace the air in a root canal with a liquid, using a square broach to pump the liquid to the apex, as a round tapering broach entering a canal of the same shape acts as a valve, preventing the air from escaping. The square broach comes in contact with the canal along its four angles, letting the air escape and the liquid takes its place. Eucalyptol compound is the liquid. This liquid is displaced by eucapercha compound, a semi-solid, followed by gutta-percha points."—H. C. Werts in *Summary.*

*The Septic Wheel-brush.* Dr. Feldman's indictment of the tooth-brush may be somewhat overdrawn, but if it is, it is on the safe side. He deserves credit for provoking discussion of that subject.

There is another brush that should be indicted and its use stopped, and that is the engine wheel-brush used by some dentists for the purpose of cleaning burs and broaches.

This rapidly revolving brush cleans (?) from the burs and broaches the filthy, septic debris that accumulates on them in their use and thoroughly distributes it in the air of the office breathed by the dentist and his patients.

A better way is to sterilize burs that are worth it and use broaches in but one case. Broaches are not expensive.

Whether a dentist uses this method or the brush-wheel is an indication of his intelligence.—R. R. C. in *Digest.*

*Sterilizable Washers for Hypodermic Syringes.* Washers to be used on hypodermic syringes are easily made out of block tin of desired thickness and are far preferable to leather washers, as they are durable and can be boiled when necessary.—M. M. Brown, *Dental Digest.*

*Emetin: A Warning.* Those using emetin solutions should be very careful not to get any in the eyes. Such an accident is followed by a very severe reaction. There is no pain at first, but in about eight hours there is an uncomfortable "scratchy" feeling, intense photophobia, and lacrimation, with conjunctival and some circumcorneal injection. Duration is about two days.—J. B. Blue. *Journal American Medical Association.*

*Rapid Disinfection of the Hands.* The *Rundschau* reprints from the *Deutsche Medizinische Wochenscrift* the description of a method of sterilizing the hands and the field of operation under adverse conditions—such as lack of pure and boiling water, in emergency cases, in war surgery, etc.—which occasionally may prove valuable to the dental surgeon. After the hands have been washed, not "scrubbed" according to surgical routine, they are treated with the following solution: Balsam of Peru 4 parts; castor oil 2 parts; Venice turpentine 2 parts; glycerin 1 part; absolute alcohol 100 parts. With this solution, which Frank calls sterolin, the hands and forearms are thoroughly rubbed with sterile swabs for two minutes, from 5 to 10 cc. of the solution to be poured on a gauze or cotton swab. Special attention is to be paid to the subungual spaces and the outer edge of the palm of the hand. After the disinfecting procedure, the mixture is allowed to dry by evaporation. It does not render the hands sticky nor does it attack them, but on the contrary renders them soft and supple. The reliability of this method has been tested in some 10,300 operations with perfect results.—R. Frank in *Zaharztlicher Rundschau,* Berlin.

*Varnish for Silicate Cement Fillings.* A good varnish for protecting silicate cement fillings against moisture may be made by dissolving sticky wax in ether.—R. S. Boys, *Commonwealth Dental Review.*

*Testing Root Canals for Cleanliness.* To a broach, which may be either barbed or smooth, is attached a pellet of cotton, dipped into hydrogen dioxide solution, and the broach is introduced into the canal, leaving it there for about thirty seconds. The broach is removed with the cotton still on it, and dipped into a receptacle, which must be perfectly clean, also containing hydrogen dioxid. If bubbles form, the canal is not clean.

When the broach with the pellet of cotton containing the $H_2O_2$ was introduced into the canal the $H_2O_2$ immediately dissolved the organic matter, which in turn was taken up by the cotton on the broach. Thus upon placing the broach into the receptacle containing the $H_2O_2$ a froth is produced. If after several applications the formation of the froth has been eliminated, the canal has reached a state of cleanliness and is ready to be filled with the proper materials.—J. A. Klein, *The Acorn.*

*Sealing Medicaments in Teeth.* A pledget of cotton saturated with thick chloro-percha is superior to cotton and sandarac for sealing medicaments in cavities.—*Dental Digest.*

*Cast Inlays.* In the casting process the result at which we aim is always the same and unchangeable. We desire an inlay that will as accurately fit the cavity as the best made foil filling. To obtain such an exact result means studying out and following a most exacting technic in a most exact manner. It means a definite and invariable rule as to cavity formation. It means a definite and invariable quality of wax. It means a definite and invariable temperature at which the wax is used. It means a definite and invariable method of removing the wax from the cavity to avoid alteration thereof. It means a definite and invariable method of investing the pattern. It means a definite and invariable method of burning out the wax. It means a definite and invariable method of casting, with a definite and invariable quality of gold. The man who thus definitely and invariably practices the casting process will obtain definite and invariable success. "Within my knowledge there are but two men in the whole world who thus definitely and invariably achieve success in casting gold inlays. Neither one of these is practicing dentistry."—Dr. Ottolenguir in *Items of Interest.*

*To Grind Natural Teeth Painlessly.* Much of the discomfort in the use of stones is occasioned by the jarring or vibration of the stone against the tooth. If the tooth is held firmly in the socket or against one wall of the socket with the thumb or finger of the left hand while grinding down enamel or opening cavities with stones it will minimize the discomfort immeasurably. Of course it is understood that all stones should run smoothly and true and that a stream of water should flow on them while cutting. If these precautions are taken, any ordinary case of grinding can be done painlessly.—E. D. in *Dental Review.*

*Necrotic Pulps.* *Under no condition should the canal of a tooth be left open for drainage of pus and gases while it is being treated.* By doing so, the operator is simply wasting his time and reinfection will certainly follow. At the first sitting, the cavity of decay in the crown of the tooth should be thoroughly cleansed of all debris and carious dentine, and the roof of the pulp chamber be removed. The removal of the contents of the canal, however, should never be attempted at this time. A treatment of formo-cresol follows, to be placed in the pulp chamber, but never forced into the canals, great care being exercised in sealing it, lest pressure be created and some of the decomposed pulp material be forced through the apex. If these directions be followed carefully, soreness rarely results, as the formo-cresol neutralizes, or prevents the formation of irritants by the micro-organisms. Even if gases or pus be present, the tooth should not be left open, but if drainage be deemed necessary it should be established by opening through the tissues at the apex of the root, an operation that may be painlessly accomplished by one of several methods that have recently come into general use.

Twenty-four hours is sufficient time to elapse after the first treatment of formo-cresol, when it should be removed.

The canals are then cleaned and washed with sterile water, a second treatment usually being applied for another twenty-four hours; this is followed by a test treatment of oil of cloves, which is allowed to remain for several days, after which the canals are filled. If the tooth does not respond after the second treatment, drainage should be established by an artificial sinus through the tissues at the apex of the root, especially if pus be present; this to be followed by the 50 per cent, sulphuric acid treatment, which consists of placing one or two drops of sulphuric acid in the canal and creating pressure by means of unvulcanized rubber until the acid is forced through the sinus. Its passage through the fistula is made known by its bubbling when it comes in contact with a quantity of bicarbonate of soda which has previously been placed at the opening of the sinus to neutralize the acid and to prevent its making its way along the gum tissue, which would destroy its external membrane.

This is followed by about two ounces of normal salt solution to cleanse thoroughly the canal and apical tissues by forcing everything before it. For this purpose I use a fine platinum point, hard soldered to the shank of a hypodermic syringe nozzle and attached to a syringe. The platinum point is forced through a piece of unvulcanized rubber into the canal as far as possible, the rubber being packed tightly into the cavity and held there. By exerting pressure on the piston, the solution is forced through the canal to irrigate thoroughly it and the sinus. This method is not advisable in the treatment of upper posterior teeth unless the maxillary sinus has been definitely located. There is no good reason for not filling the roots immediately after this treatment, for if the condition be not cured it will require surgical interference, hence further treatment through the canals would be useless. If trouble follows this method of treatment the root filling should not be removed but the tooth should be extracted, or the operation of removing the apex of the root and all adjacent necrotic tissue should be resorted to.—Dr. J. F. Biddle, in March *Items of Interest.*

*A Better Gold Inlay.* The cavity should be prepared with walls diverging slightly more than for cast inlays. All enamel margins should be left sharp and well defined.

The cavity should be moistened or oiled and an impression taken with warmed modeling compound. The compound is then chilled and carefully removed and examined to see if all margins are clearly recorder in the impression. A bite in wax is then taken and patient dismissed.

A die of amalgam is made from the impression and by the aid of wax bite mounted on an anatomical articulator. From the amalgam die is taken any number of impressions in modeling compound until accuracy is assured. A cement die is then made and after being separated from the impression is invested in either modeling compound or plaster to strengthen the mass and protect any frail walls.

Gold foil is now to be packed into the cavity by hand pressure only and tooth restored to desired contour and occlusion.

Filling may be removed from cement die and tried in the amalgam die from time to time to secure size, contour, etc.

Great care is to be exercised during packing against margins and quite a surplus should be used. A flat gold-burnisher is then to be used to aid in condensing the entire surface, following the rule of burnishing toward and not away from margins.

When filling is completed the cement die with filling in place is thoroughly dried out and then heated in a Bunsen flame until it assumes a cherry-red color.

The inlay when cool is placed in amalgam die properly seated and given final trimming, shaping and polishing except at margins where a fine feather edge is to be left. Inlay is now ready to be inserted for try-in in the mouth and if found correct (it will be correct if preparation of cavity and impression were correct) it is removed and preparations made for cementing. Depending upon the case, undercuts may or may not be necessary.

A thin, smooth mix of a good inlay cement is used after sterilizing and drying cavity and inlay is inserted to place with considerable hand pressure. *Do not use a mallet.* While the cement is still soft burnish the margins. The final finish may be given at this or a subsequent sitting. Use only very fine abrasives and avoid strips and discs as much as possible. The burnisher properly used for final finishing is the ideal instrument.

The result will be a gold inlay with margins nearer perfect than I believe possible to obtain with any other filling material or process. The gold is harder than a well condensed gold filling, yet soft enough to be easily manipulated at margins without evil results.

Of the advantages of this method it might be stated that for large restorations it is much easier for the patient and less tiresome for the operator with the added advantage that the inlay is practically finished when inserted, requiring only a final burnishing of margins.

By the use of this method where large gold fillings are indicated, more satisfactory operations will result and much time and energy will be saved.—W. G. Sherman, in *Digest*.

*Treatment of Pyorrhea.* Take resublimed iodine, C. P. carbolic acid (crystal), make a saturate solution. It usually takes about three weeks' standing to get a good solution, and the longer it stands the better it gets, as the solution improves with age.

Wash the pus pockets around the teeth well with peroxide of hydrogen, put in the iodine preparation (with a wooden pointed instrument) and flow over it, tannate of glycerine; then allow the saliva of the mouth to flow over the tannate of glycerine, thus forming a tannate of albumen, as a protection to the iodine treatment.

Repeat the treatment at intervals of about forty-eight hours until two or three treatments of the iodine solution have been made; then prescribe a mouth-wash to be used daily, night and morning, of the following formula:

Carbolic acid, five drops; compound tr. iodine, ten drops; tannate of glycerine, one drachm; water sufficient to make eight ounces.

You will find that after the first application of the iodine treatment, all pus will have disappeared, and there will be a marked difference in the appearance of the gums. In three or four weeks' time, if this treatment is followed up carefully, all symptoms of Riggs' disease or pyorrhea alveolaris (if you prefer) will have disappeared, never to return again, provided the patient will follow your instructions to keep his or her mouth clean. In the majority of your patients you will find the greatest difficulty of affecting a permanent cure; when once relieved they will become careless in the care of their teeth, unless you can impress them with the necessity of keeping them clean.—Dr. G. Chisolm, *Dental Items of Interest.*

*Separating Modeling Compound Impressions.* In taking modeling compound impressions, the compound may easily be separated from the cast if the impression is painted with a thin solution of shellac before it is poured. A most perfect impression may be obtained if the compound be vaselined and held under a stream of hot water for a few seconds just before the impression is taken.—R. Davis in *Dental Review.*

